# Effect of Culture Conditions on the Production of an Extracellular Protease by *Bacillus* sp. Isolated from Soil Sample of Lavizan Jungle Park

**DOI:** 10.4061/2011/219628

**Published:** 2011-12-07

**Authors:** Abbas Akhavan Sepahy, Leila Jabalameli

**Affiliations:** ^1^Department of Microbiology, North Tehran Branch of Islamic Azad University, 1667934791 Tehran, Iran; ^2^Department of Biology, Science and Research Branch of Islamic Azad University, 1477893855 Tehran, Iran

## Abstract

Soil samples of Tehran jungle parks were screened for proteolytic *Bacilli*. Among eighteen protease producers one of the isolates obtained from Lavizan park, in north east of Tehran, was selected for further experimental studies. This isolate was identified as *Bacillus* sp. strain CR-179 based on partial sequencing of 16S rRNA. Various nutritional and environmental parameters affected protease production by *Bacillus* sp. strain CR-179. Protease production by this *Bacillus* cultivated in liquid cultures reached a maximum at 24 h, with levels of 340.908 U/mL. Starch and maltose were the best substrates for enzyme production while some pure sugars such as fructose, glucose, and sucrose could not influence production of protease. Among various organic nitrogen sources corn steep liquor, which is commercial, was found as the best substrate followed by yeast extract, whey protein, and beef extract. The optimal pH and optimal temperature of enzyme production were 8.0 and 45°C, respectively. Studies on enzymatic characterization revealed that crude protease showed maximum activity at pH 9.0 and 60°C, which is indicating the enzyme to be thermoalkaline protease.

## 1. Introduction

Proteases are one of the most important industrial enzymes and are used in a variety of industrial applications, such as laundry detergents, pharmaceutical industry, leather industry in dehairing and bating of hides, manufacture of protein hydrolyzates,food industry like meat tenderizing, cheese flavour development, treatment of flour in the manufacture of baked goods, improvement of dough texture, flavour and colour in cookies, and so forth [1–6], silver recovery from X-ray films [7], and even in waste processing industry [8, 9]. These enzymes account for about 60% of the total enzyme market [10–12]. Microbial proteases are preferred to enzymes from plant and animal sources, since they possess almost all the characteristics desired for biotechnological applications [13]. Commercial proteases are mostly produced from various bacteria, and it was reported that about 35% of the total microbial enzymes used in detergent industry are the proteases from bacteria sources [14]. Among bacteria *Bacillus* sp. are specific producers of extracellularly proteases [15] and can be cultivated under extreme temperature and pH conditions to give rise to products that are, in turn, stable in a wide range of harsh environments [16]. Furthermore, many *Bacillus* sp. secrete large amounts of proteases than that required for their physiological activities [17]. The cost of enzyme production is a major obstacle in its successful industrial application [18], so it should be produced in high yields in a low-cost medium.

In the present study we report the isolation of alkaline protease producer* Bacillus* sp. strain CR-179, from soil sample of Lavizan jungle park, and then focus on optimizing the production of extracellular protease by testing various environmental and nutritional factors.

## 2. Materials and Methods

### 2.1. Microorganism

Soil samples were taken from Tehran jungle parks included Lavizan, Chitgar, Sorhke hesar, Taleghani, and Khojr. The isolated bacteria were inoculated on to skim milk agar plates and incubated at 37°C for 24–72 h. Appearance of clearing zones formed by hydrolysis of skim milk revealed the capability of bacteria for producing protease. The protease producers were then subcultured on to nutrient agar plates in order to obtain pure isolates of bacteria species. The resulting isolated colonies were subcultured on to nutrient agar slants, grown at 37°C for 24 h, maintained at 4°C, and subcultured at four-week intervals. They were identified as *Bacillus* species based on gram staining, cellular morphology, and some biochemical tests. Genetical analysis was also done for one of the species. For sequencing analysis, the genomic DNA was extracted from the isolate, using Roche kit. The amplification of the16S rDNA was performed through PCR technique, using Taq DNA polymerase, genomic DNA as a template, and 3′forward and 5′ reverse universal primers. The primers used have nucleotide sequence as

3 f: 5′ - AGAGTTTGATCCTGGC-3′,
5 r: 5′- TACCTTGTTACGACTT-3′.

PCR products were sent to SQ lab Co. (Germany). By receiving the results, the 16S rDNA nucleotide sequence of isolate has been deposited in GenBank and aligned with the 16S rRNA sequences available in nucleotide database in NCBI, (National Center for Biotechnology Information, Available at: http://www.ncbi.nlm.nih.gov/), using BLAST software, (Basic Local Alignment Search Tool) [19].

### 2.2. Protease Production

The culture medium used in this work for protease production contained (g/L of distilled water): corn steep liquor 4.0, starch 10.0, KCl 0.3, MgSO_4_ 0.5, K_2_HPO_4_ 0.87, and CaCl_2_ 0.29. The pH was adjusted to 7.0-8 with 1% Na_2_CO_3_, and this medium was sterilized by autoclaving at 121°C for 15 min. The above medium (50 mL in 250 mL Erlenmeyer flasks) was inoculated with 1 mL of an overnight culture and incubated at 45°C in a rotary shaker operated at 150 rpm for 24 h. At the end of fermentation period, the contents were centrifuged at 15500 g for 15 min at 4°C, and the cell-free supernatant was used as crude enzyme for enzyme assay.

### 2.3. Protease Assay

The activity of protease was assessed in triplicate by measuring the release of trichloroacetic-acid soluble peptides from 0.5% (w/v) casein in 50 mM glycin NaOH (pH 9.0) at 60°C for 10 min. The 1 mL reaction was terminated by adding 0.5 mL of 10% trichloroacetic acid. It was left for 15 min and then centrifuged at 14000 g for 10 min. One unit of enzyme activity was defined as the amount of enzyme required to release 1 *μ*g of tyrosine/min under standard conditions [20].

### 2.4. Effect of Culture Components on Protease Production

The effect of carbon sources 1% (w/v) and nitrogen sources 0.4% (w/v) on enzyme production was determined by growing the isolate in production media with different carbon and nitrogen sources. In this study various carbon sources such as glucose, galactose, maltose, lactose, starch, sucrose, and fructose were used. Sources of nitrogen included yeast extract, beef extract, corn steep liquor, whey protein, peptone, tryptone, and urea.

### 2.5. Effect of pH on Protease Production

The effect of pH on protease production was determined by growing the isolate in production media with an initial pH range of 6 to 10 using 1% Na_2_CO_3_.

### 2.6. Effect of Agitation Rate on Protease Production

The effect of agitation rate on enzyme production was investigated by incubating culture flasks at different agitation speed of 110, 130, 150, and 180 rpm.

### 2.7. Effect of pH on Protease Activity

The optimum pH for enzyme activity was determined with casein 0.5% (w/v) as substrate dissolved in different buffers (sodium phosphate, pH 6-7, Tris-HCl, pH 8-9, and glycine NaOH, pH 9–11).

### 2.8. Effect of Temperature on Protease Activity

The effect of temperature on enzyme activity was determined by performing the standard assay procedure at pH 9 within a temperature range from 40°C to 75°C.

## 3. Results and Discussion

**Figure 1:**
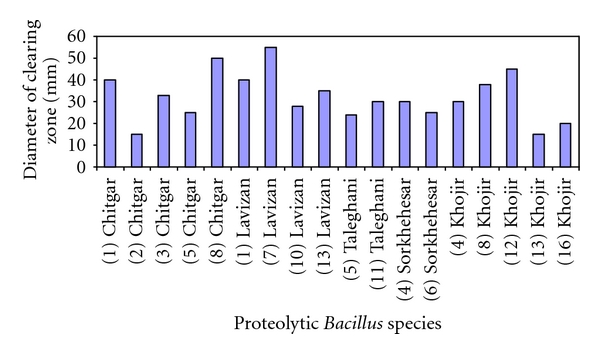
Diameter of the clear zones of proteolytic *Bacillus* species on skim milk agar.

Members of the genus *Bacillus* are widely used in industry in the large-scale production of enzymes, such as proteases. Of particular industrial importance are proteases with activity at alkaline pH and high temperature [21]. In the present study forty bacterial isolates were obtained from soil samples of which eighteen isolates were identified as proteolytic* Bacillus* species based on gram staining, cellular morphology, and some biochemical tests such as lecithin, gelatin, and casein hydrolysis in which all the species were positive in these tests. The proteolytic activity was assayed using skim milk agar and expressed as diameter of clear zones in mm. One of the *Bacillus* isolated from Lavizan jungle park (L7) exhibited the highest proteolytic activity with a clear zone diameter of 55 mm after 72 h, although other isolates from soil samples of Khojir and Chitgar parks exhibited clear zone diameter of 45 and 40 mm, respectively.  Figure 1 compares clear zones of proteolytic *Bacillus* species from different soil samples on skim milk agar.

The isolate L7 was selected for further experimental studies in order to optimize the production of protease. The BLAST search of 16S rRNA gene sequence against sequences in nucleotide database has shown 97% homology with *Bacillus* sp. strain CR-179 16S rRNA gene sequence with accession number of AJ82128.

### 3.1. Culture Conditions for Enzyme Production

**Figure 2:**
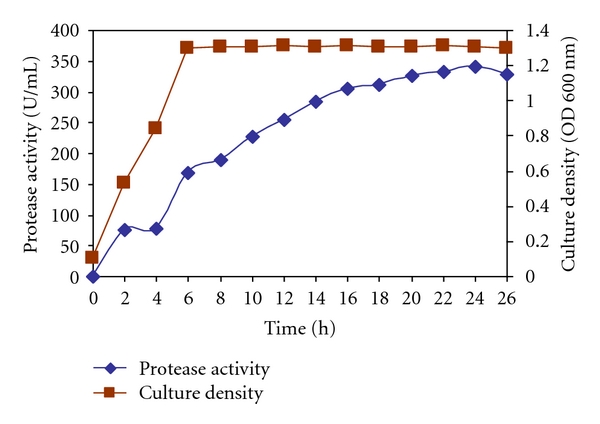
Protease production as a function of cultivation time by *Bacillus* sp. strain CR-179 grown on starch (1%) and corn steep liquor (0.4%) in shake flasks at initial pH 8 and at 45°C.

Figure 2 reports the time course of protease production by *Bacillus* sp. strain CR-179 in liquid medium containing starch (1%) as carbon source and corn steep liquor (0.4%) in 250 mL erlenmeyer flasks. The formation of protease significantly started from early stationary phase and reached a maximum in 24 h, with levels of 340.980 U/mL and then began to fall. In a similar study maximum protease activity was determined at the 18th hour, which occurred in the late stationary phase, when most of the bacteria sporulated [22]. *Bacillus *sp. are spore-forming bacteria; thus during sporulation and also germination,it increases protease activity [23]. Scientists acclaimed that during sporulation and germination, hydrolyzed proteins were used to compose proteins for endospores or vegetative cells [24, 25]. This process requires an increase of protease production. This is in contrast to previous report which showed that *Bacillus* sp. usually produce more protease during the late exponential phase [26]. Another investigation done by Asokan, S. and Jayanthi, C. [27] revealed different results; they observed that the optimum incubation time for enzyme production is 96 hours.

In an earlier investigation maximum activity of protease was attained after 48 hours of fermentation, after which the activity started to decline [28].

Using of cost-effective growth medium for the production of alkaline proteases from an alkalophilic *Bacillus* sp. is especially important [20]. Therefore, there is a need to find new strains of bacteria with the ability of producing proteolytic enzymes with novel properties and the development of low-cost media. The present investigation was aimed at optimization of medium components which have been predicted to play a significant role in enhancing the production of alkaline proteases [29]. *Bacillus* sp. strain CR-179 was capable of using a wide range of carbon sources, but production of protease varied according to each carbon source (Table 1). In the present study starch was the best substrate for enzyme production, followed by maltose, while glucose, fructose, and sucrose were less effective. Moderate to good amount of protease activity was produced in the presence of lactose and galactose. This is in agreement with previous report which showed that starch caused high level of enzyme expression in *Bacillus* species [30]. But this is in contrast to a recent report which showed that maltose and starch caused low protease production [31].

Utilization of maltose and galactose was also shown to result in better growth than consuming starch, although starch was the best substrate for enzyme production. It suggests that optimum conditions for protease production are not necessarily the same as the best conditions for growth. This observation is in contrast to previous study done by Camila Rocha da Silva et al. They showed that starch was the best carbon source for both growth and protease production [32].

Organic nitrogen sources such as corn steep liquor, beef extract, yeast extract, whey protein, tryptone, and peptone were tested on the growth and protease production of *Bacillus *sp. (Table 2).

Results obtained revealed that corn steep liquor, which is commercial, led to both maximum protease production and growth. So by using corn steep liquor in the growth medium, the production cost of the enzyme can be lessened. In some organisms, however, organic nitrogen sources were found to be better nitrogen sources both for growth and also protease production [33, 34]. In a similar study Singh et al. used cheap nitrogen sources such as corn steep liquor for the production of a thermostable acid protease by a strain of *Aspergillus niger* F2078 [35]. Although in this study other organic nitrogen sources such as yeast extract, beef extract, and whey protein could result in good amount of enzyme production, they were less effective than corn steep liquor. This finding is in contrast to Shafee et al. They found beef extract as the best substrate for protease production [30]. In an earlier report Uyar et al. found skim milk to have significant effect on the production of the extracellular protease [36].

Protease activity varied with initial pH of the culture medium (Table 3). The highest levels of protease activity were detected in the cultures grown at pH 8. The growth was the highest at pH 8 too. It suggests that *Bacillus* sp. strain CR-179 can be classified as alkaliphilic *Bacilli,* since alkaliphiles are defined as organisms that grow optimally at alkaline pH, with pH optima for growth being in excess of pH 8 and some being capable of growing at pH > 11 [37, 38]. In a similar study Das and Prasad considered the pH of 8.0 as the best pH for protease production [39].

Microorganisms vary in their oxygen requirement. In particular, O_2_ acts as a terminal electron acceptor for oxidative reactions to provide energy for cellular activities. The variation in the agitation speed has been found to influence the extent of mixing in the shake flasks and also affect the nutrient availability [40]. Agitation rates have been shown to affect protease in various strains of bacteria [41, 42]. In the present investigation*, Bacillus* sp. strain CR-179 grown in culture media containing starch and corn steep liquor showed maximum protease activity at 150 rpm agitation speed after 24 h incubation (Table 4). At this speed, aeration of the culture medium was increased which could lead to sufficient supply of dissolved oxygen in the media [43]. Nutrient uptake by bacteria also will be increased [44] resulting in increased protease production. At 180 rpm protease activity was found to be reduced. This was perhaps due to denaturation of enzymes caused by high agitation speed [45]. High agitation rates could also damage bacterial cells, so that reduction of protease producers will result in decreased protease production. Agitation speed of 110 and 130 rpm affected the growth of the organism considerably. At this agitation rates, insufficient aeration and nutrient uptake perhaps caused the inability of bacteria to grow efficiently. In a similar study a notable increase in the protease production with the high agitation rate (>200 rpm) was reported. It was also revealed that a decrease in agitation rate drastically lowered the total protease yield [46].

### 3.2. Effect of pH on Protease Activity

**Figure 3:**
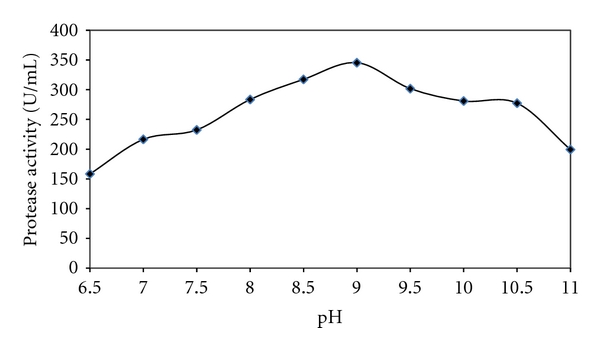
Optimum pH profile of the *Bacillus* sp. strain CR-179 protease grown at 45°C for 24 h.

From an industrial prospective, the protease must exhibit considerable activity at high pH (s) and temperature (s). A pH range between 6.5 and 11.0 was used to study the effect of pH on protease activity (Figure 3). The crude protease had a relatively wide pH range between 8.0 to 10.5, with maximum enzyme activity at pH 9.0. Proteases that have pH optima in the range of 8.0 −11.0 are grouped under the category of alkaline proteases [47–49]. Maximum activity at pH 9.0 indicates the enzyme to be alkaline protease. Hence this protease can be introduced as an industrially and economically feasible enzyme. In a similar study Ibrahim et al. found an alkaline protease which had maximum activity at pH 10.0 [50].

### 3.3. Effect of Temperature on Protease Activity

**Figure 4:**
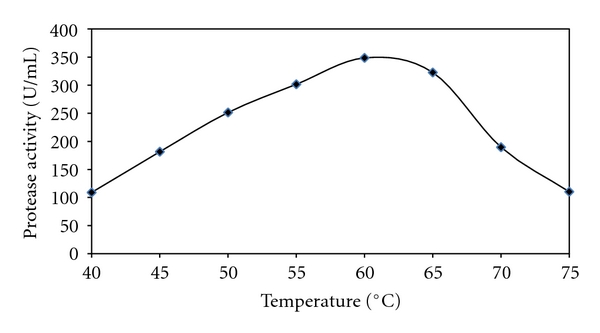
Temperature profile of the *Bacillus* sp. strain CR-179 protease grown at 45°C for 24 h.

The protease activities were assayed at different temperatures ranging from 40°C to 70°C at a constant pH of 9.0 (Figure 4). Enzyme activity increased with temperature within the range of 40°C to 60°C and showed reasonable activity at temperature range of 50°C–65°C, with maximum activity at 60°C. A reduction in enzyme activity was observed at values above 60°C. Maximum activity at 60°C indicates the enzyme to be thermoprotease. This observation was similar to the report of Nascimento et al. They worked on a thermophilic *Bacillus* sp. which produced a protease with maximum activity at 60°C [40]. Maximum protease activity at high temperatures is a very suitable characteristic for its industrial acceptability.

## 4. Conclusion

In the present investigation we report the isolation of proteolytic *Bacilli* from Tehran jungle parks. As the characterization and optimization for each factor of growth, nutritional requirement, and production yields are essential requirements before the selected strain is used for further investigation, the yield improvement of alkaline protease, in general by any microbial system, depends on the physiological, nutritional, and biochemical nature of the microbe employed, and these factors vary from organism to organism [14, 51–53]; one of the strongest isolates*, Bacillus* sp. strain CR-179, was selected in order to optimize the culture conditions for protease production. Scientists preferred studying new isolates because they could be alternative for commercial use [54–56]. This strain can be used for large-scale production of alkaline protease to meet the present day demand of the industrial applications. Starch and corn steep liquor are cheap carbon and nitrogen sources which led us to propose a low-cost medium formulation for maximum protease production. Major industrial units are continuously trying to identify enzymes that have potential industrial applications, either to use them correctly or to create popular enzymes with enhanced catalytic activity for well-adapted large-scale industrial processes [57]. The optimum pH and temperature for enzyme activity were determined as 9.0 and 60°C, respectively. As the thermoactivity and pH stability of proteases are of great importance in industrial uses, enzymatic properties indicate the potential use of this bacterium and its protease for various industrial applications.

## Figures and Tables

**Table 1:** Effect of carbon source on *Bacillus* sp. strain CR-179 growth and protease activity. The culture density and extracellular protease activity determined during 24 h incubation at 45°C and initial pH 8.

Carbon source	Culture density (OD 600 nm)	Maximum enzyme activity (U/mL)
Starch	1.428	337.532
Lactose	1.322	229.740
Fructose	1.125	76.493
Galactose	1.512	297.272
Maltose	1.620	303.766
Glucose	1.121	52.597
Sucrose	1.117	49.740

**Table 2:** Effect of nitrogen source on *Bacillus* sp. strain CR-179 growth and protease activity. The culture density and extracellular protease activity were determined during 24 h incubation at 45°C and at initial pH 8.

Nitrogen source	Culture density (OD 600 nm)	Maximum enzyme activity (U/mL)
Corn steep liquor	1.410	336.233
Whey protein	1.114	239.870
Beef extract	1.383	203.246
Yeast extract	1.391	308.961
Tryptone	0.954	190
Peptone	1.030	119.350
Urea	0.666	46.883

**Table 3:** Effect of pH on growth and protease activity by *Bacillus* sp. strain CR-179 cultivated in a liquid medium containing starch (1%) and corn steep liquor (0.4%) in shake flasks during 24 h at 45°C.

Initial pH	Culture density (OD 600 nm)	Maximum enzyme activity (U/mL)
6	0.980	168.18
7	1.335	260.129
8	1.402	335.454
9	1.012	253.37
10	0.734	163.506

**Table 4:** Effect of agitation rate on growth and protease activity by *Bacillus* sp. strain CR-179 cultivated in a liquid medium containing starch (1%) and corn steep liquor (0.4%) in shake flasks during 24 h at 45°C.

Agitation rate (rpm)	Culture density (OD 600 nm)	Maximum enzyme activity (U/mL)
110	1.327	301.688
130	1.388	320.389
150	1.418	345.844
180	1.330	307.142
